# Tamm–Horsfall protein in humane urine: sex-dependent differences in the excretion and *N*-glycosylation pattern

**DOI:** 10.1038/s41598-023-44650-1

**Published:** 2023-10-19

**Authors:** Boris Mo, Birte Scharf, Christian Gutheil, Matthias C. Letzel, Andreas Hensel

**Affiliations:** 1 Institute of Pharmaceutical Biology and Phytochemistry, University of Münster, Münster, Germany; 2 Organisch-Chemisches Institut, University of Münster, Münster, Germany

**Keywords:** Biochemistry, Urology

## Abstract

Tamm–Horsfall protein (THP) is a highly *N*-glycosylated protein from epithelial cells of the ascending limb of Henle loop. It is secreted into the urine as part of the innate immune response against uropathogenic pathogens. As women are more likely to suffer from urinary tract infections, biomedical studies were conducted to investigate sex-differences in THP excretion, as well as differences in the THP *N*-glycosylation pattern. A total of 238 volunteers (92 men, 146 women, 69 with hormonal contraceptives) participated in this study, providing urine samples. Women showed a clear tendency to have higher THP concentration and excretion rates than men (*p* < 0.16). Regular intake of hormonal contraceptives had no significant influence on urinary THP concentration compared to no regular intake. The individual *N*-glycosylation pattern of THP in urine samples from randomly selected individuals (10 female, 10 male) was investigated after enzymatic release and MS analysis of the oligosaccharides. Female subjects tended to have an increased proportion of oligomannose type *N*-glycans and non-fucosylated glycans, whereas men had an increased proportion of fucosylated complex-type glycans. The higher level of oligomannose-type glycans in THP from women might be explained by a self-defence mechanism to overcome the higher infections pressure by the female anatomical properties.

## Introduction

Tamm-Horsfall Protein (THP, syn. Uromodulin) is the most abundant protein found in humane urine and has first been described in 1952 by Igor Tamm and Frank Horsfall during the search for inhibitors of viral hemagglutination^1^.

THP is produced for more than 90% by the epithelial cells of the thick ascending limb of the loop of Henle in the kidney and to a lesser extent (about 10%) by epithelial cells of the early part of the distal convoluted tubule^2^. The molecular mass of THP has been determined to be in the range of 90 to 100 kDa by SDS-PAGE, representing a highly glycosylated protein with 616 amino acids, including 48 cysteine residues involved in 24 disulfide bridges^3^. THP has 8 potential *N*-glycosylation sites (Asn38, Asn76, Asn80, Asn232, Asn275, Asn322, Asn396, and Asn513)^4^. Considering its amino acid sequence, three additional epidermal growth factor-like (EGF) domains are predicted^4^. THP also shows a domain (D8C) with an unknown function and a two-part *zona pellucida* module (ZP-N/ZP-C) which is essential for polymerization and the formation of filaments under physiological conditions^4^. These THP-filaments are capable of binding pathogens such as uropathogenic *E. coli* (UPEC)^4–6^. Within the endoplasmatic reticulum the polypeptide part of the protein is processed towards a glycoprotein with high degree of oligomannose type *N*-glycans side chains, which are subsequently matured in the Golgi into complex glycans consisting of *N*-acetylglucosamine, fucose, galactose and sialic acid which are predominantly arranged in antenna-like structures. It has previously been reported that the *N*-glycan at position Asn275 is not further modified by the Golgi and therefore remains as an *N*-glycan of the oligomannose type^4,7,8^.

Due to the high degree of oligomannose type *N*-glycans, THP interacts with mannose-binding proteins, e.g. the mannose-sensitive subunit FimH of type 1 fimbriae from UPEC, which correlates with a faster elimination of these pathogens from infected tissue or from the urine^4^. This has also been proven by THP knockout mice, which are more prone to urinary tract infections (UTI) caused by UPEC with type 1 fimbriae, while P-fimbriae are not affected by THP^9^.

Beside the innate immune defence against infections of the urinary tract, THP is also known to influence the formation of kidney gravels or stones and the progression of urolithiasis, as the negative charge of sialic acid-containing oligosaccharide side chains of THP interacts with calcium oxalate or calcium phosphate by precipitation^10,11^. A clear correlation between the formation of kidney stones and THP deficiency has been observed in THP knockout mice^12,13^. The ability of THP binding to cations is crucial in order to prevent urolithiasis and the prevalence of non-infectious chronic bladder disease, and within different studies also the glycosylation pattern of THP has been described to be essential for the specific cation binding properties^10,14–16^.

UTI are one of the most common infectious diseases worldwide, caused in about 80% of all cases by UPEC, but also *Pseudomonas aeruginosa*, *Klebsiella pneumonia* (about 7%), *Proteus mirabilis* (5%), *Enterobacter cloacae*^17^, *Enterococcus faecalis*, *Streptococcus bovis* or *Candida albicans* contribute to the high prevalence of UTI. The incidence of UTI is about five times higher for women, compared to men^18^. Main risk factors are sex, age, history of UTI and healthcare-associated UTI catheterization^19,20^. Antibiotics are used as standard treatment for UTI, but antibacterial resistance and high recurrence rates emphasize the importance to develop alternative preventive strategies. Adhesion inhibitors, interacting with the early host–pathogen interaction inhibit the bacterial attachment to the host cells, as shown for example for specific FimH inhibitors^21,22^ or by interfering with the fimbrial assembly^23^. Also probiotics^24^ or vaccination^25^ could be options for UTI treatment in near future.

Interestingly, a recent investigation of an extract from cranberry fruits (*Vaccinium macrocarpon*) indicated a new mechanism for modulation of the innate immune defence against UPEC, namely the stimulation of THP in the kidney, resulting in significant elevated THP levels in urine samples of volunteers after 7 day of oral intake of cranberry extract^26^. The increased THP level correlated with significantly reduced bacterial adhesion of UPEC to human bladder cells when cultivated in urine samples of cranberry treated subjects^27^. Interestingly, *V. macrocarpon* extract increased the THP secretion in urine only in male volunteers, while women were not influenced by the intervention^26^. This had been the first report on THP inducing exogenous compounds, and the observed effect was described to be sex-specific. Follow-up studies and screening for further THP inducers indicated that seven day oral intervention with an aqueous horsetail herb extract (*Equisetum arvense*), a plant-based medication used traditionally for treatment of uncomplicated UTI, significantly increased THP excretion in both male and female volunteers^28^. Both datasets revealed a sex-dependent effect. In addition, women are also more likely to suffer from UTI^19,29^, therefore, further investigations were needed to elucidate differences in THP secretion in male and female volunteers.

As previously reported, pregnancy^30^ and genetic defects^31^ are capable of changing THP concentration in urine as well of influencing the THP glycosylation pattern. Based on these reports the question arose, if basal THP concentrations between male and female subjects might be different, and if the THP titers in urine are affected by the hormonal status, e.g. during intake of hormonal contraceptives. Also, the question on the *N*-glycosylation pattern of THP in regard to sex has still not been investigated. Therefore, the aim of the following study was to investigate potential differences in basal THP concentration between men and women, and to investigate the detailed N-glycosylation in regard to the respective sex.

## Results and discussion

### Sex-dependent differences in urinary THP-excretion

THP concentrations in urine samples were quantified within two independent biomedical studies. Study population 1 (biomedical study 2019–177-f-S; Table 1) was conducted in 2019 with 178 healthy volunteers in total (age 19 to 72 years, average 27.2 years) including 62 men (35%) and 116 women (65%). 34% (61 individuals) of the female volunteers reported regular intake of hormonal contraceptives (group designation: F + C). For confirmation of the results of study (1) a further independent study (2) was performed in 2021 (biomedical study 2021-084-f-S; Table 1) recruiting 60 healthy volunteers (age 20 to 37 years, average 25.3 years), including 30 female and 30 male subjects. It was decided to evaluate both studies separately due to differences in sample collection: Morning midstream urine had not been collected in study population (1), but was collected in study population (2). For reasons of acceptance of the biomedical studies by the ethical committee it was not possible to collect 24 h urine from the volunteers (which would give more precise data), instead only one time sampling was allowed.

**Table 1:** THP, log_10_(THP), creatinine and log_10_(THP/creatinine) values obtained from the biomedical study (1) 2019-177-f-S, biomedical study (2) 2021-084-f-S and combined analysis of (1) and (2), including subgroup-analysis.

	n	Ø THP [µg/mL] ± SD (Median)	Creatinine mg/dL ± SD (Median)	ØLog_10_THP (µg/mL) ± SD	ØLog_10_([THP µg/Crea mg]) ± SD
Study population (1)					
Total volunteers	178	9.5 ± 10.5 (5.4)	84.1 ± 60.9 (63.5)	0.77 ± 0.42	0.96 ± 0.67
Male (M)	62	7.6 ± 7.9 (4.5)	110.3 ± 71.8 (104.0)	0.67 ± 0.41	0.75 ± 0.66
Female (F)	116	10.5 ± 11.6 (5.8)	69.8 ± 48.4 (54.5)	0.83 ± 0.41	1.08 ± 0.64
Without hormonal contraceptives (F–C)	61	11.7 ± 13.3 (5.3)	60.9 ± 41.3 (54.5)	0.87 ± 0.42	1.17 ± 0.63
With hormonal contraceptives (F + C)	55	9.1 ± 9.3 (6.2)	81.1 ± 53.3 (66.7)	0.78 ± 0.41	0.99 ± 0.64
Study population (2)					
Total volunteers	60	13.3 ± 12.06 (9.0)	138.1 ± 69.2 (134.9)	0.94 ± 0.43	0.87 ± 0.49
Male (M)	30	12.7 ± 11.0 (8.9)	154.6 ± 61.6 (150.9)	0.89 ± 0.48	0.74 ± 0.47
Female (F)	30	13.9 ± 12.9 (9.0)	121.5 ± 72.5 (110.3)	0.99 ± 0.36	0.99 ± 0.48
Without hormonal contraceptives (F–C)	16	12.2 ± 11.1 (8.6)	94.3 ± 50.9 (82.9)	0.93 ± 0.38	1.02 ± 0.47
With hormonal contraceptives (F + C)	14	15.9 ± 14.5 (10.0)	152.7 ± 80.5 (155.2)	1.06 ± 0.35	0.95 ± 0.49
Study population (1 + 2)					
Total volunteers	238	10.5 ± 11.1 (6.1)	97.7 ± 67.3 (80.1)	0.81 ± 0.4	0.92 ± 0.6
Male (M)	92	8.9 ± 8.9 (5.7)	124.6 ± 71.7 (125.0)	0.74 ± 0.4	0.73 ± 0.6
Female (F)	146	11.4 ± 12.2 (6.7)	80.5 ± 58.0 (63.5)	0.86 ± 0.4	1.03 ± 0.6
Without hormonal contraceptives (F–C)	77	12.0 ± 12.9 (6.1)	68.0 ± 45.6 (58.5)	0.88 ± 0.4	1.12 ± 0.6
With hormonal contraceptives (F + C)	69	10.7 ± 11.5 (7.1)	95.8 ± 66.5 (76.3)	0.83 ± 0.4	0.94 ± 0.6

**Figure 1:**
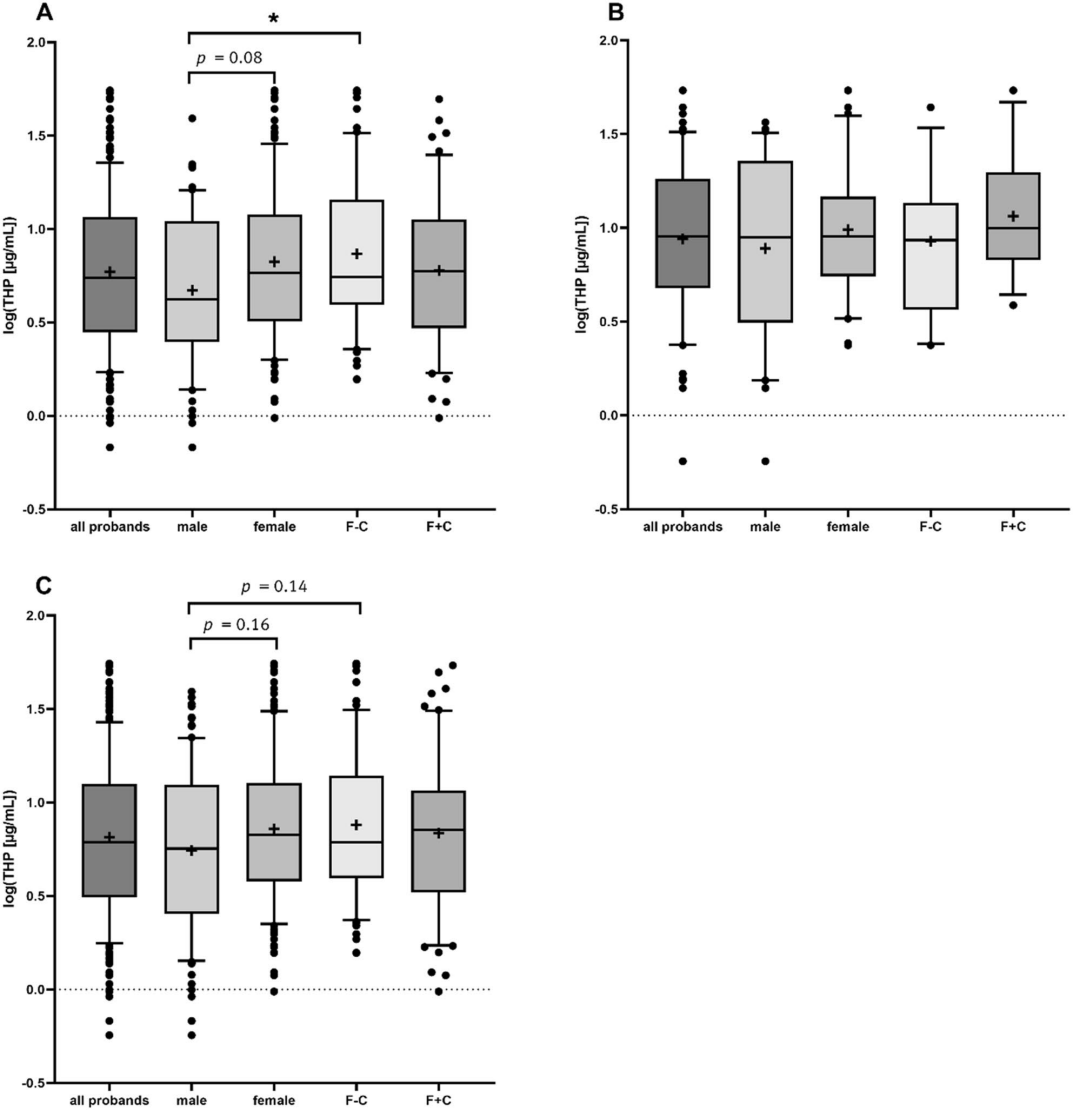
(**A**) Log_10_(THP [µg/mL]) of study population (1) from biomedical study 2019–177-f-S. (**B**) Log_10_(THP [µg/mL]) of study population (2) from biomedical study 2021–084-f-S. **C** Log_10_(THP [µg/mL]) of combined study populations (1 + 2) from biomedical studies 2019 177 f. S and 2019 177 f. S. Subgroups: male subjects; female subjects; F–C: female subjects with no regular hormonal contraceptive intake; F + C: female subjects with regular hormonal contraceptives intake. Box plots depict the 10 to 90 percentile, + represents mean value. Data was processed by one-way ANOVA. Subsequently, post-hoc test was conducted by Tuckey’s multiple comparison. *p < 0.05.

As depicted in Table 1 and Fig. 1 A1/2 the average THP concentration in urine samples is 9.5 μg/mL. No significant differences in THP concentrations between male and female volunteers were detected due to high interindividual variability, but a tendency to higher THP values in the female group was obvious (7.6 ± 7.9 vs. 10.5 ± 11.6 µg/mL, *p* = 0.24). This tendency gets more pronounced when using the respective log_10_(THP) values (Table 1 and Fig. 1, *p* = 0.08), indicating a trend of men producing less THP than women. Interestingly, female subjects without any intake of hormonal contraceptives (F–C) showed tendencies towards higher THP values compared to women using hormonal contraceptives F + C (log_10_(THP) 0.87 ± 0.42 vs. 0.78 ± 0.41), although no statistical significance was determined. Statistical siginificane was observed for comparison of the male subgroup with the F–C subgroup (0.67 ± 0.41 vs. 0.87 ± 0.42, *p* < 0.05), while comparing the men vs. F + C, did not reveal statistical differences.

Further evaluation was performed by normalisation of the obtained THP values to the individual creatinine concentrations in the urine (THP excretion; THP_ex_) (Supplementary Data, Figure S1 and Table 1). Significant differences in log(THP/Crea) between male and female individuals were observed (0.75 ± 0.7 vs. 1.08 ± 0.6, *p* < 0.05). For unambiguous evaluation of these data it has to be kept in mind that creatinine-standardized values for comparison of sex differences of biomarkers are critical as creatinine itself shows sex difference, and its urinary level is higher in male than in female due to differences in muscle mass. For this reason, the creatinine normalized data should not be over-interpretated, but in the case here described, they can be used to gain insight in the THP excretion itself. Further analysis on creatinine concentration only, showed—as expected—significant differences between male and female subjects (110.3 ± 71.8 vs. 69.8 ± 48.4, *p* < 0.0001) (Table 1). Comparison between F–C and F + C revealed no statistical significance, but clear tendencies towards elevated creatinine values in F + C (60.9 ± 41.3 vs. 81.1 ± 53.3, *p* = 0.33).

Evaluation of biomedical study (2) revealed similar trends for THP concentration and the creatinine-normalized THP as found in study (1). (Table 1 and Fig. 1B, Supplementary Data Figure S1). Again men had lower THP (12.7 ± 11 vs. 13.9 ± 12.9 µg/mL) and log_10_(THP) (0.89 ± 0.5 vs. 0.99 ± 0.4 µg/mL) in comparison to women Comparison between F–C and F + C in this study population again shows different urinary THP levels (12.2 ± 11.1 vs. 15.98 ± 14.6 µg/mL).The tendencies for higher THP values for women compared to men is also reflected by the respective creatinine-normalized concentrations.

To further validate the observations, the data sets from both biomedical studies (1) 2019–177-f-S and (2) 2022–084-f S were combined and analysed for urinary THP and creatinine. The combined dataset included 238 participants, 92 men (39%) and 146 women (61%) of whom 69 (29%) were regularly using hormonal contraceptives (F + C subgroup). As shown in Table 1 and Fig. 1C male volunteers showed lower urinary log_10_(THP) levels in comparison to female (0.74 ± 0.4 vs. 0.86 ± 0.4 µg/mL, *p* = 0.16). Again, this tendency is also reflected by the respective creatinine-normalized THP concentrations. Subgroup analysis indicated, as expected, non-significant lower THP levels of the F + C group compared to F–C (0.83 ± 0.4 vs. 0.88 ± 0.4 µg/mL).

To summarise the combined data set analysis, differences in log_10_(THP) are observed between the subgroups male ~ female (p = 0.16) and male ~ F–C (*p* = 0.14): Male volunteers exhibited lower log_10_(THP) than women, and hormonal contraceptive intake has a negative influence on log_10_(THP). This tendency is also clearly reflected by evaluation of the creatinine-nominalised THP values (Supplementary Data, Fig. S1).

In summary, differences in THP and THP_ex_ between men and women are observed within two independent biomedical studies. There is also a tendency for men to have lower levels of THP in urine, but simultaneously higher levels of creatinine. This result is reflected by evaluation of both, the absolute THP (Table 1, Fig. 1) concentration as well as the creatinine-normalized values (Supplementary Data, Figure S1). The reason for these differences in THP secretion or expression is not clear. Mechanistical investigation using THP-secreting cells are difficult to perform, as the cultivation of primary THP^+^-cells from renal distal tubular cells is known to be challenging and THP-secreting cell lines are not available up to now. The regular intake of hormonal contraceptives seems to have an influence on the renal metabolic activity, as not only a lower amount of THP in the urine was observed, but also an increase in the creatinine level in comparison with no regular use of contraceptives is obvious. As it is known that the prevalence of UTI is higher in women in comparison to men^30^ mainly due to anatomical differences, an upregulation of THP in women could indicate an increase of self-defence mechanism due to the more frequent exposure to uropathogenic pathogens in women. Data provided from this study showed similarities to previously published studies conducted by the *Swiss Kidney Project on Genes in Hypertension* (SKIPOGH) and *Cohorte Lausannoise* (CoLause), in which THP was investigated as a marker for tubular functionalities^32^. Further studies are needed to elucidate the effect of the intake of hormonal contraceptives in women on the THP excretion.

### *N*-glycosylation pattern of THP from male and female urine samples

**Figure 2:**
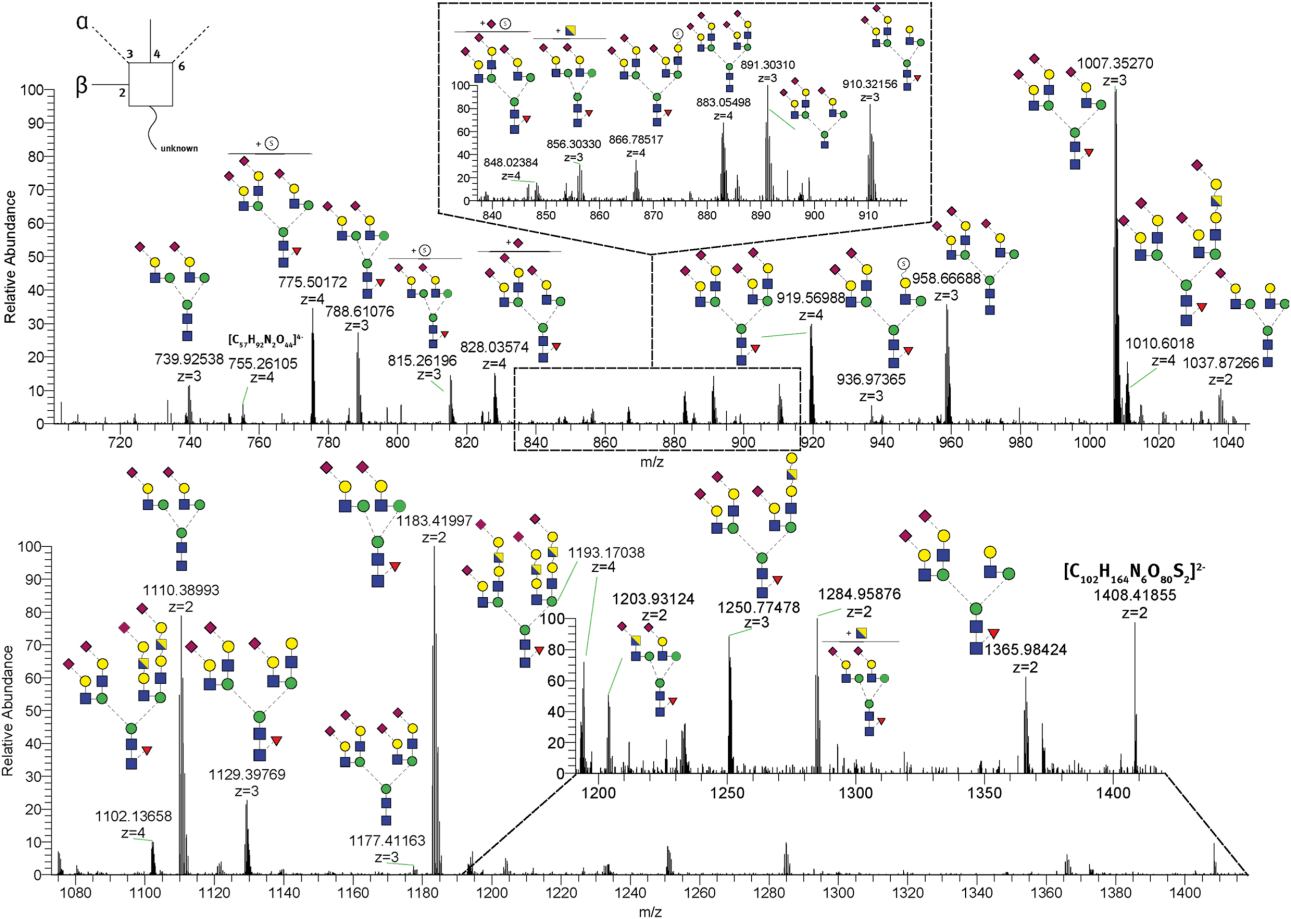
Example of a mass spectrum from high resolution (-)ESI analysis of *N*-glycans released from human THP, obtained from a urine sample of a male volunteer. Selected masses and the proposed oligosaccharides are based on database search (GlycoMod). In total, 56 *N*-glycans were identified. Linkages and isomers are depicted according to previously published literature^34^.

The *N*-glycosylation pattern of THP was investigated according to a modified protocol of Patabandige et al. (2020)^33^. In principle THP was isolated from the urine samples by 80 kDa centrifugal filtration, followed by denaturing of the protein, enzymatic release of the *N*-glycan oligosaccharides by use of PNGase F and subsequent high resolution mass spectrometric analysis (SDS-PAGE of THP enriched from urine samples after 50 kDa centrifugal filtration and after PNGaseF treatment is displayed in the Supplementary Data File, Figure S2). Identification of the glycans was based on a data base search (GlycoModTool) by using the respective *m*/*z* values, the respective isotopic mass and isotope pattern. A typical high resolution mass spectrum and the proposed oligosaccharides are displayed in Fig. 2.

Table S1 in the Supplementary Data File summarizes the detailed structural features of 56 oligosaccharides identified to be part of THP.

As sex-dependent differences in THP secretion have been found, and also gender-dependent differences in *O*-glycosylation of THP have recently been reported^30^, differences in *N*-glycosylation between male and female volunteers are likely and therefore to be investigated. The THP *N*-glycosylation pattern was analyzed in 10 samples of freshly (same day of collection and THP isolation) collected individual morning midstream urine from 10 individuals (5 male and 5 female, average age 25.2 years) and the individual *N*-glycosylation pattern was analyzed. Additionally, morning midstream urine from another 10 volunteers (5 male, 5 female, average age 26.4 years) was investigated, but these urine samples were analyzed after the liquid had been stored frozen for several weeks at -20 °C. Therefore, the N-glycosylation pattern was analyzed separately for the “fresh” samples and for the “frozen, stored” samples.

**Figure 3:**
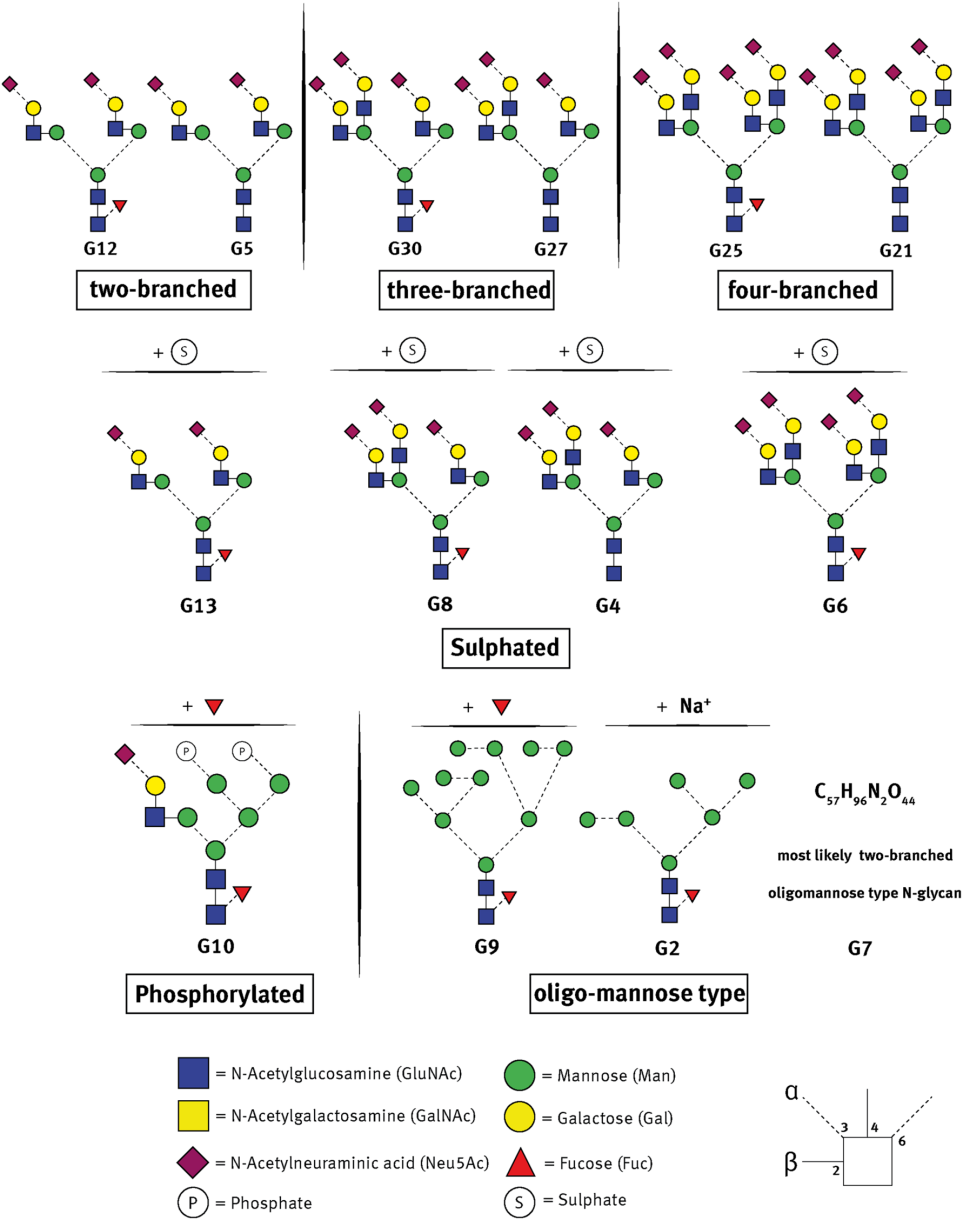
Selected N-glycans from THP used for relative quantification. Glycan ID (Gx) were chosen according to the occurrence of the species in the mass spectra (m/z-sorted). The selected glycans represent approximately 80% of the peak area fraction in the spectra in the 600 to 2000 m/z range. Different types of N-glycans were chosen: G12 and 5 (two-branched); G30 and 27 (three-branched); G25 and 21 (four-branched); G13/8/4/6 (sulphated); G9/2/7 (high-mannose type) and G10 (phosphorylated). Determination of the linkage between the *N*-glycans was based on GlycoMod database. Molecular structure of G7 could not further be specified, as molecular mass indicated the presence of a pentose, which is unlikely in mammals.

For relative quantitative evaluation, 14 different glycans, displayed in Fig. 3, representing different *N*-glycan types (two/three/four-branched, fucosylated, sulphated, phosphorylated, high mannose type) were used. From these selected 14 oligosaccharides 7 glycans (G12/5/30/27/25/21/8) represent approximately 80% of the total peak area fraction in the spectra in the 600 to 2000 m/*z* range.

**Figure 4:**
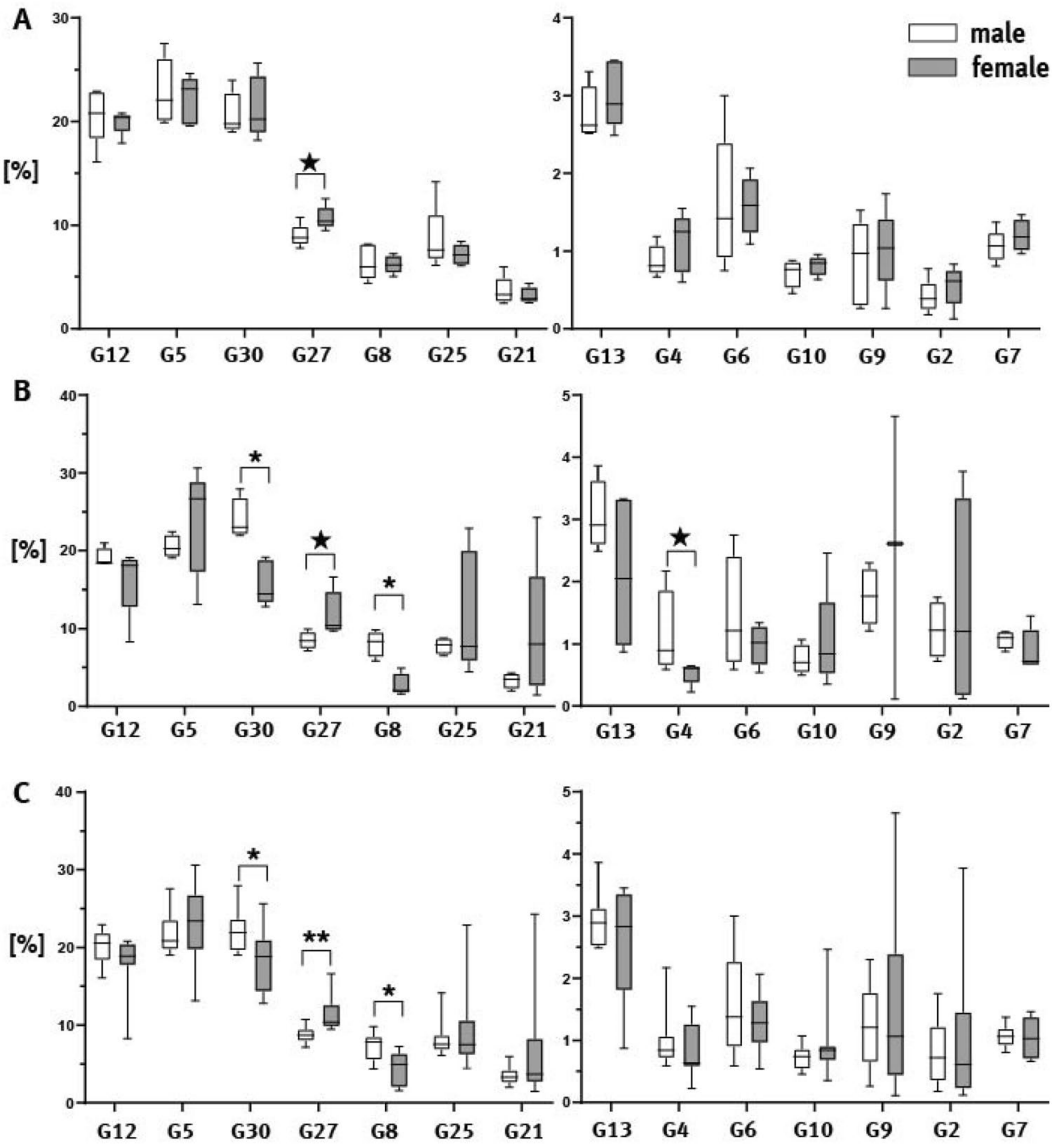
Gender-dependent differences in THP N-glycosylation pattern. Quantification was based on the relative intensity of selected glycan species (G12, 5, 30, 27, 8, 25, 21 [left] and G13, 4, 6, 10, 9, 2, 7 [right]. (**A**) Glycosylation analysis of THP from freshly obtained urine samples (same day of sample collection and THP isolation) from 5 male (white bars) and 5 female (black bars) untreated volunteers. (**B**) Glycosylation analysis of THP from the biomedical study 2021-084-f-S (5 male and 5 female). (**C**) Grouped analysis of the glycans of both freshly obtained urine and day 0 untreated control. Statistical analysis was performed for each glycan with Mann–Whitney test (non-parametric T-test): *p < 0.05, **p < 0.001 and ⭐ 0.15 > *p* > 0.05.

The relative quantification data of the 14 selected THP *N*-glycans from urine samples from male and female volunteers is shown in Fig. 4. Freshly collected morning midstream urine (Fig. 4A, 5 male, 5 female) showed a high degree of similarity between the individual samples and between the male and female urine samples. Only for the oligosaccharide **G27**, a three-branched, non-fucosylated complex type oligosaccharide, significant tendencies were found between men and women (10.7% ± 1.02 *vs* 8.9% ± 0.96, *p* = 0.056). **G27** was found at higher concentrations in women than in urine samples from men.

Analyses of the “stored” samples (Fig. 4B) also revealed sex-differences for **G27**, but interestingly, also differences in three other three-branched oligosaccharides **G30**, **G8** and **G4** were detected. This might indicate changes in the glycosylation pattern during storage.

Combining the data sets from the two investigations with n = 20 samples (10 male and 10 female) indicated significant differences. **G30**, **G8** and **G27** showed (highly) significant differences in the relative quantities. Male exhibited higher quantities in **G30** (three-branched fucosylated complex-type) (18.3% ± 3.3 vs. 22.5% ± 2.8) and **G8** (three-branched fucosylated, sulphated complex-type) (4.6% ± 2.3 vs. 7% ± 1.5), which are both fucosylated, three-branched glycans. Female volunteers showed significantly elevated relative quantities of **G27** (three-branched non-fucosylated complex-type) (10.4% ± 2.5 vs. 9.7% ± 1.5).

From these data it can be concluded that a high degree of similarity in *N*-glycosylation pattern of THP from men and women is obvious, but 3-branched oligosaccharides, especially **G27,** might be subjected to a certain kind of sex-variability. As glycan modification may lead to different pharmacological and also physico-chemical properties of THP^35–37^ , further investigations on the *N*-glycan of THP is necessary to understand functionality changes of the protein after modification: As sialic acid and sulfate modifications are important for the isoelectric point of THP and also for the binding of multivalent cations, THP *N*-Glycosylation modifications due to genetic mutations may lead to the increase of kidney stone formation and other related diseases which has been shown also for podocalyxin, an important glycoprotein from the kidney^16,38–40^. Concerning potential sex related differences in the glycosylation pattern or inter-individual differences in expression of glycosyltransferase of THP have been reported. Phenotypic differences for the β-D-1,4-*N*-acetylgalactosaminyltrasferase 2 have been previously described^41^. Interestingly, this enzyme is expressed as a function of age and in different parts of the kidney^42^. Detailed investigation on mRNA expression of this or similar enzymes in tubular cells are missing but might be a promising research issue.

The relative glycan quantification is a robust and strong method to compare glycan composition between independent groups and/or individuals on THP *N*-glycosylation but also for other glycoproteins, as discussed recently by Patabandige et al.^33^. The authors showed that the MS analysis and the sample preparation were robust, due to non-significant differences in signal intensities between replicates. Fortunately, independent replicates also showed similar results^33^. Within their study, the following glycans showed the highest relative signals: **G25 > G27 > G5 > G12 > G30** (G1, G7, G20, G21, G29 in their publication), while for our glycan study, the following glycans showed the highest relative signals: **G5 > G12 > G30 > G27 > G25**. There is a different order of signal intensity (due to different equipment and modified sample preparation steps), but the highest 5 signals of both datasets are congruent. Therefore, obtained data from our study can substantially be confirmed by the investigation of Patabandige et al*.*^33^.

From these data, differences in the fine structure of THP are obvious and future studies should be performed to correlate potential differences in glycosylation pattern to different functional aspects of THP.

## Methods

### THP-quantification (ELISA)

THP concentration in individual and pooled urine was determined by an in-house sandwich-like ELISA^43^, modified as described recently^44^.

One hundred microliter of a solution (10 µg/mL) of wheat germ agglutinin from *Triticum vulgaris* (Sigma-Aldrich, Taufkirchen, Germany), diluted in coating buffer (pH 9.6, Na_2_CO_3_ 50 mM, NaHCO_3_ 349 mM, NaN_3_ 0.02% (w/v) in H_2_O), was used for coating of 96-well plates (Nunc Maxisorp; Thermo Fisher, Dreieich, Germany) for 2 h at RT, while gently shaking. After rinsing 3 × with washing buffer, containing Tween 20, 0.05% in PBS, blocking of non-specific binding sides was performed by use of 200 µL blocking buffer (2% BSA in washing buffer) for 2 h at RT. After incubation, plates were rinsed 3 × with washing buffer. Residual buffer was removed by upside down centrifugation (1 min, 50 × g) on an absorbent paper. Coated plates were sealed and stored at − 20 °C (maximum 1 week) if not used immediately. Urine samples (100 µL) were diluted 1:20 or 1:40 (50 µL or 25 µL in 1 mL) with blocking buffer (1:40, in cases were absorption at λ = 450 nm was out of the calibration range). The diluted samples (100 µL) were added to the pre-coated wells and incubated for 2 h at RT, while gently shaking. Blocking buffer served as blank. External calibration standards and diluted samples were run in duplicate. After incubation, plates were washed 3 × with washing buffer. In-between each washing step, residual buffer was collected in a container and then centrifuged upside down on an absorbent paper (1 min, 50 × *g*,) to ensure complete removal of residual buffer. Sheep-anti-Human-Tamm-Horsfall Glycoprotein (BioRad, Feldkirchen, Germany) (100 µL, 1: 10,000 in blocking buffer) were added into each well and incubated for 2 h at RT, while gently shaking. After the incubation, the plates were washed as described above. 100 µL of rabbit anti-IgG (H + L)-HRP conjugate (BioRad, Feldkirchen, Germany) (1:3000 in blocking buffer) was dispensed and incubated for 1 h at RT, while gently shaking. The plate was washed again. For photometric detection, 100 µL of 3,3′,5,5′-tetramethylbenzidine (TMB Liquid Substrate System for ELISA, Sigma-Aldrich, Taufkirchen, Germany) were added and incubated under shaking for 10 min in the absence of direct light. After adding stop solution (100 µL of sulfuric acid 4 mol/L), measurements were performed immediately at λ = 450 nm with λ = 550 nm as reference wavelength. Intensity of photometric absorbance is directly proportional to THP concentration present in samples. Standards were plotted into a four-parameter logistic curve fit (log_10_[concentration]) to calculate the respective THP concentration in the urine samples.

### Biomedical study (2019-177-f-S)

The biomedical study “Qualitative and quantitative analysis of Tamm-Horsfall protein in human urine samples” was approved by the ethics committee of University of Münster (Ethik-Kommission der Ärztekammer Westfalen-Lippe, Germany), acceptance code 2019–177-f-S. The research was performed in accordance with relevant guidelines/regulations. Informed consent was obtained from all participants.

Midstream urine samples were anonymously submitted at the pharmacy department (University of Münster) between June and August 2019. Containers provided for urine acquisition were consecutively labelled by numbers. Volunteers had to fill a questionnaire after each urine sampling. The following data were requested: number of the sample, age, sex, and the use of hormonal contraceptives. Anonymity was ensured due to anonymised sample submission. In total, 178 individuals (19 to 72 years; median 24 years; average 27.2 years, 63 males [35%], 115 females [65%], of whom 60 [33%] were taking hormonal contraceptives) gave written informed consent on collecting their own midstream urine. For data evaluation, the study was divided into four subgroups (male, all female, female with or without intake of hormonal contraceptives). Exclusion criteria included antibiotic therapy two weeks prior to the study as well as the intake of drugs, affecting renal and hepatic excretion rate six weeks prior to the study. Pregnant and breastfeeding women were not included in the study. The respective urine samples were used for quantification of THP by a validated in-house ELISA ≥ 2 independent quantifications with n = 2 technical replicates) and creatinine was determined by Münster University Hospital. Urine samples were stored at − 20 °C, aliquots at − 80 °C prior to analysis. Declaration of consent is kept under seal at the Institute for Pharmaceutical Biology and Phytochemistry.

### Biomedical study (2021-084-f-S

“THP concentrations in urine samples”) was performed as described in detail^28^ and was approved by the ethics committee of University of Münster Ethik-Kommission der Ärztekammer Westfalen-Lippe, Germany), acceptance code 2021-084-f-S. The research was performed in accordance with relevant guidelines/regulations. Informed consent was obtained from all participants.

### *N*-Glycosylation pattern of THP

Enrichment of THP by centrifugal filter units was performed as previously described with slight modifications^33,45^: Precipitate and particles in urine samples were removed by centrifugation (1.000 × *g*, 10 min). Enrichment of THP from native urine was performed with pre-rinsed centrifugal filter units (MWCO 50 kDa, Pierce Protein Concentrator PES 50). 10 mL of urine were transferred into the filter units and the filters were centrifuged in a swinging bucket for 15 min (3.000 × *g*, 4 °C). The enriched protein fraction was washed 2 × with 15 mL of cold LC–MS grade water. The pellet was transferred into 1.5 mL micro reaction tubes and dried in an evaporation chamber.

Reduction and alkylation of THP: The pellet was dissolved in 200 µL of a mixture of 6.0 M guanidinium hydrochloride, 250 mM trizma base/HCl and 65 mM dithiothreitol, pH 8.6, to reduce cysteine bridges. After 60 min (56 °C, occasional stirring) 2.5 mg of iodoacetamide were added and the mixture was incubated for 45 min under exclusion of light.

Desalting by SEC cartridges was performed using Sephadex G-25 columns (NAP-5, GE Healthcare, Sweden), which had been equilibrated prior to use: Excess packaging buffer was removed and the columns were equilibrated by 4 × 2.5 mL of 50 mM NH_4_HCO_3_ buffer. Reduced and alkylated proteins were loaded. 500 µL of NH_4_HCO_3_ buffer were added and eluted. Eluted buffer was discarded. Another aliquot of buffer (500 µL) was added to the column. The respective flow through was collected into a micro reaction tube. Desalted protein fractions were dried by evaporation. Subsequently, ammonium carbonate buffer was removed by adding 250 µL dH_2_O. The solution was dried by 2 × evaporation.

Glycan release and enrichment: Dried and desalted glycoproteins were dissolved in 200 µL dH_2_O and 2 µL of PNGase F solution (2 µg/mL in dH_2_O; PNGase F, recombinant, Serva, Heidelberg, Germany) were added and incubated for 37 °C for 24 h. 200 µL of MeOH were added to stop the enzymatic activity. Released glycans and the residual protein fraction were separated by pre-rinsed Amico Ultra 0.5 centrifugal filter unit (10 kDa MWCO; 12.000 g, 15 min). 100 µL of the obtained released N-glycan fraction were diluted with 900 µL MeOH for MS analysis.

### Mass-based identification of oligosaccharide by Orbitrap Exploris

ESI measurements were performed on an Exploris 120 (Thermo-Fisher Scientific, Bremen). The instrument was equipped with a loop inlet (into a methanol stream). Raw (-)ESI MS data were evaluated with Thermo Xcalibur 3.1 QualBrowser. Measured *m*/*z* values of glycan species were utilised to identify released N-glycans. Calculated molecular masses were utilised to further determine glycan composition with GlycoMod tool (Expasy)^46^. The following settings were used:

**Table:** 

Experimental masses	Monoisotopic mass, as [M] (neutral)
Mass tolerance	5 ppm (0.005 mDa) up to 10 ppm (0.01 mDa)
Form of N-linked oligosaccharide	Free/PNGase released oligosaccharides
Number of Hexose	3–12
Number of HexNAc	2–12
Number of Deoxyhexose	0–3
Number of NeuAc	0–8
Number of NeuGc	0
Number of pentose	0–2
Number of sulphates	0–6
Number of phosphates	0–6

Structural composition was proposed by comparison to existing databases (Expasy GlyConnect). The sum formula was then simulated to identify detected glycan species. Mass tolerance was set to 0.005 m/*z*. After PNgaseF treatment, glycans were found as 1-amino-GlcNAc or GlcNAc derivative, resulting in a mass shift of ~ 1 Da (NH_2_ is substituted with OH).

### Relative quantification of N-glycans

Selected N-glycans (G12, 5, 30, 27, 25, 21, 13, 8, 4, 6, 10, 9, 2, 7) were utilized for relative quantification as previously described^33^. For the three most abundant highest isotope signals of each glycan intensities were summed up (= 100%) and the respective proportions were calculated. Selected glycans represented around 80% of the signals detected in the range of 600–2000 m/*z*.

### Supplementary Information

Supplementary Information.

## Data Availability

The datasets used and/or analysed during the current study available from the corresponding author on reasonable request.
