# Supplementary material for: Tamm–Horsfall protein in humane urine: sex-dependent differences in the excretion and N-glycosylation pattern
Source: Sci Rep. 2023 Oct 19;13:17815. doi: 10.1038/s41598-023-44650-1 (PMC10587112; doi:10.1038/s41598-023-44650-1)
Supplement: Supplementary file 1 — Supplementary Information. [file 41598_2023_44650_MOESM1_ESM.docx]

**SUPPLEMENTARY DATA**

**Tamm-Horsfall protein in humane urine: Sex-dependent differences in the excretion and *N*-glycosylation pattern**

**Boris Mo ^1^, Birte Scharf ^1^, Christian Gutheil ^2^, Matthias C. Letzel^2^ & Andreas Hensel^1,*^**

^1^ Institute of Pharmaceutical Biology and Phytochemistry, University of Münster, Münster, Germany

^2^ Organisch-Chemisches Institut, University of Münster, Münster, Germany

**Correspondence**

Prof. Dr. Andreas Hensel, Institute of Pharmaceutical Biology and Phytochemistry, University of Münster, Münster, Germany

Phone: +49 251 8333381, Fax: +49 251 83 38341

email: [ahensel@uni-muenster.de](mailto:fabian.herrmann@uni-muenster.de)


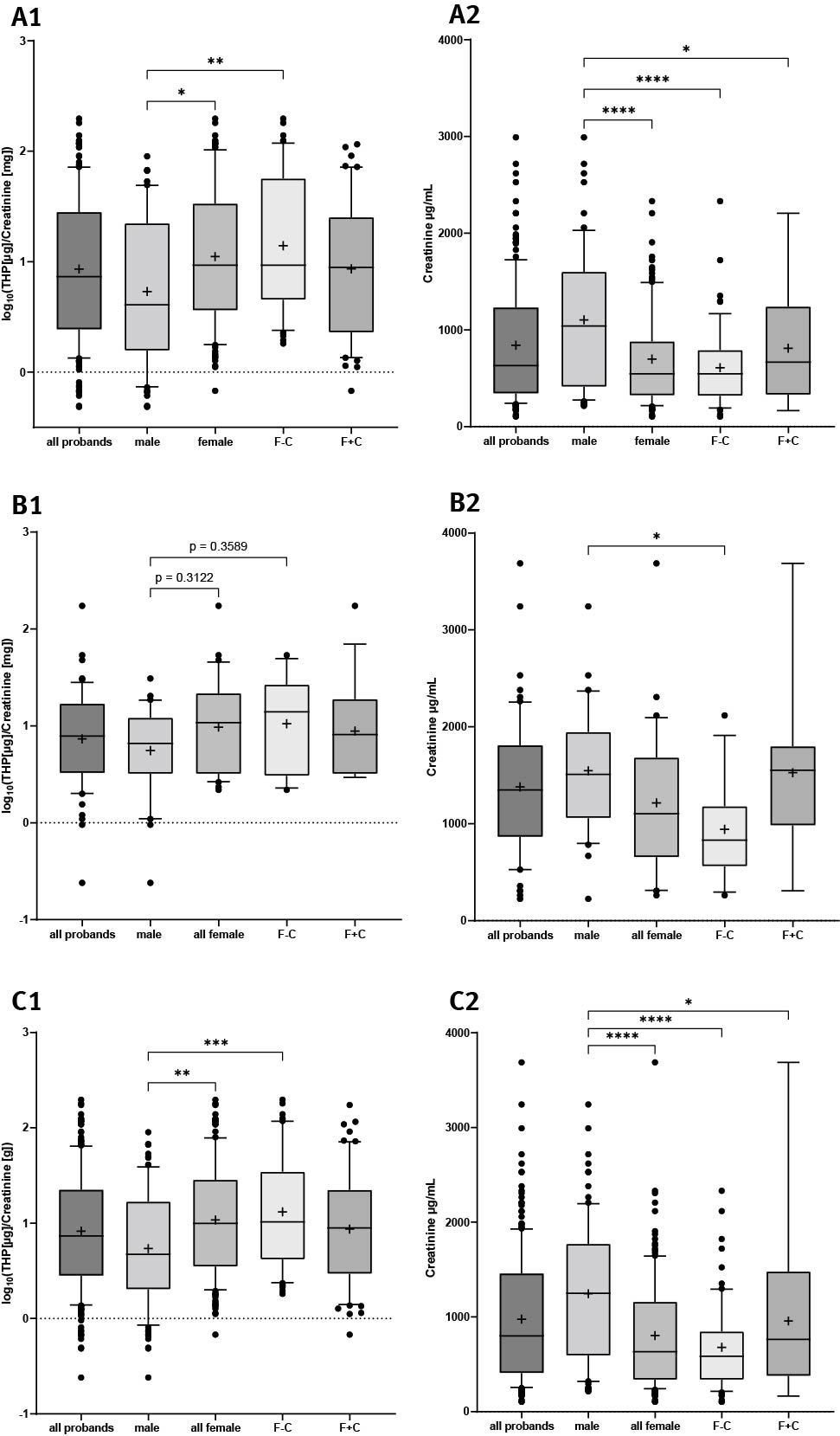


**Figure S1: A1** Log_10_(THP [µg]/Crea [mg]) of study population (1) from biomedical study 2019‑177‑f‑S and **A2** creatinine concentration in µg/mL. **B1** Log_10_(THP [µg]/Crea [mg]) of study population (2) from biomedical study 2021‑084‑f‑S and **B2** creatinine concentration in µg/mL. **C1** Log10(THP [µg]/Crea [mg]) of combined study populations (1 + 2) from biomedical studies 2019 177 f S and 2019 177 f S. **C2** creatinine concentration in µg/mL. Subgroups: male subjects; female subjects; F-C: female subjects with no regular hormonal contraceptive intake; F+C: female subjects with regular hormonal contraceptives intake. Box plots depict the 10 to 90 percentile, **+** represents mean value. Data was processed by one-way ANOVA. Subsequently, post-hoc test was conducted by Tuckey’s multiple comparison. * p < 0.05; ** p < 0.01; *** p < 0.002, **** p < 0.0001.


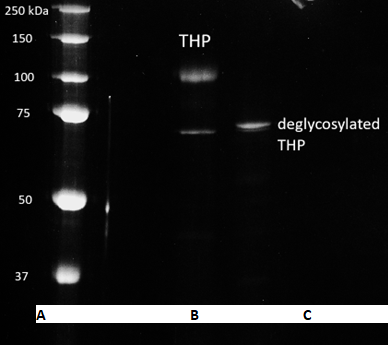


**Figure S2:** SDS-PAGE (4 to 15 % gradient gel, 80 V, 120 min) of THP enriched from urine samples after 50 kDa centrifugal filtration (lane B) and after PNGaseF treatment (lane C). Staining was performed with colloidal Coomassie Brilliant Blue G250.

**Table S1:** N-glycan species obtained by the treatment of THP with PNGaseF. In total, 56 different glycan species were detected and >90 % were identified according to database search and exact mass. Glycan code: H = hexose; N = N-acetylated glucosamin or galactosamin; F = fucose; P = Pentose; S = Sialic acid; G3S = Sulphate; Phos = Phosphate.

| **Glycan ID** | **Measured *m*/*z*** | ***z*** | **Calculated isotopic mass of [M] in Da** | **Glycan composition** | **Ion formula** | **suggested structure**  **(Based on database)** |
| --- | --- | --- | --- | --- | --- | --- |
| **G1** | **678.21896** | **5** | **3396.134** | **H6N5F1S4[G3S]1** | **[C_126_H_200_N_9_O_95_S]^5-^** | 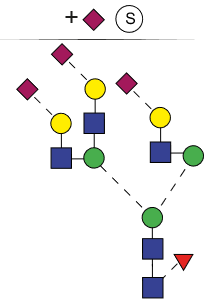 |
| **G2** | **707.73864** | **2** | **1395.51103** | **H6N2** | **[C_52_H_86_N_3_NaO_40_]^2-^** | 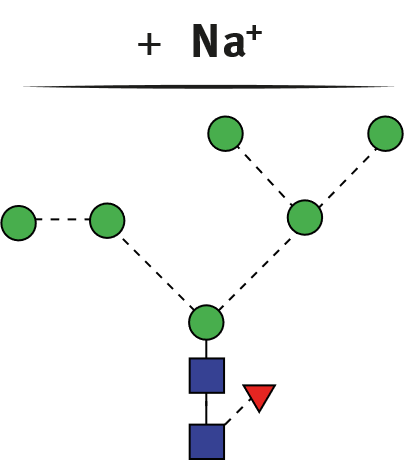 |
| **G3** | **723.90036** | **3** | **2174.7246** | **H6N4F2[G3S]1** | **[C_80_H_131_N_4_O_62_S]^3-^** | 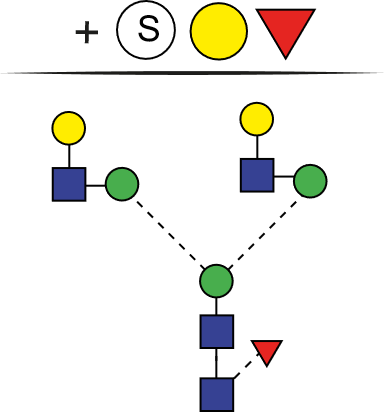 |
| **G56** | **737.59232** | **3** | **2215.8005** | **H7N3F2** | **[C_84_H_138_N_3_O_64_]^3-^** | 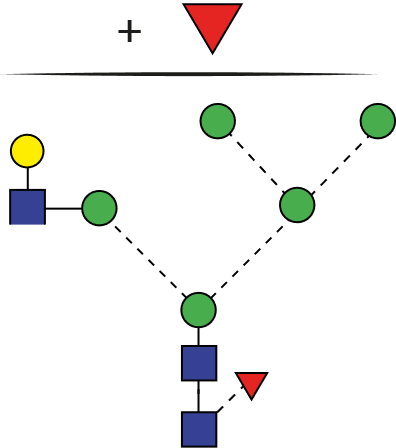 |
| **G4** | **738.73766** | **4** | **2958.982** | **H6N5S3[G3S]1** | **[C_109_H_175_N_9_O_82_S]^4-^** | 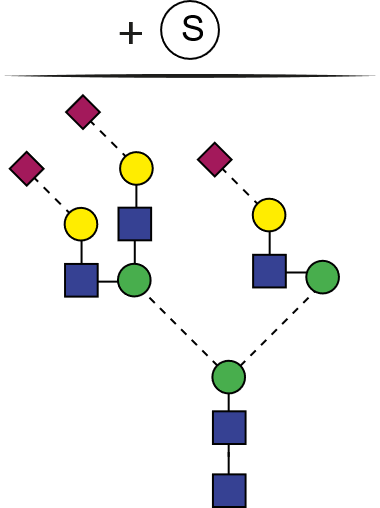 |
| **G5** | **739.59189** | **3** | **2221.79919** | **H5N4S2** | **[C_84_H_136_N_7_O_61_]^3-^** | 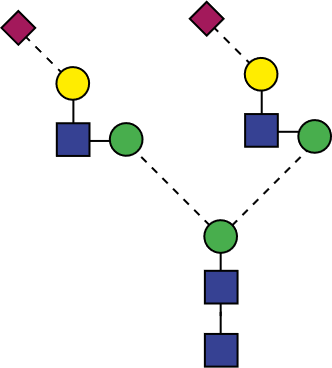 |
| **G6** | **751.2446** | **5** | **3761.2622** | **H7N6F1S4[G3S]1** | **[C_140_H_228_N_10_O_105_S]^5-^** | 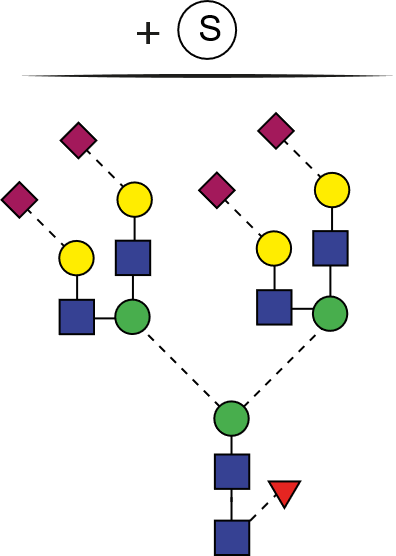 |
| **G7** | **755.26116** | **2** | **1512.538** | **H5N2F1P1** | **[C_57_H_94_N_2_O_44_]^2-^** | **Not defined** |
| **G8** | **775.25102** | **4** | **3105.03544** | **H6N5F1S3[G3S]1** | **[C_115_H_184_N_8_O_87_S]^4-^** | 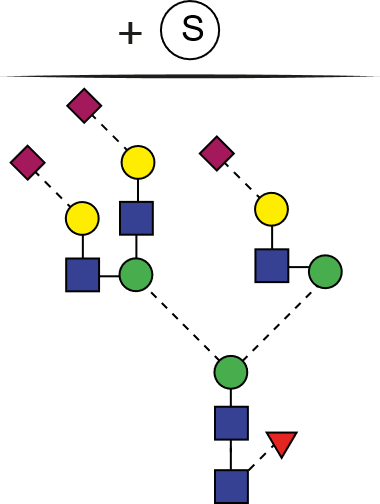 |
| **G55** | **777.23689** | **4** | **3112.97892** | **H7N6S2[G3S]2** | **[C_112_O_88_H_180_N_8_S_2_]^4-^** | 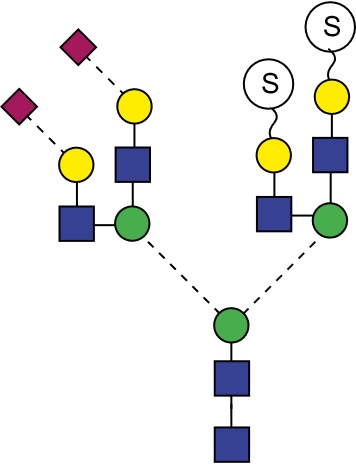 |
| **G9** | **777.93437** | **3** | **2336.82663** | **H10N2F2** | **[C_88_H_145_N_2_O_69_]^3-^** | 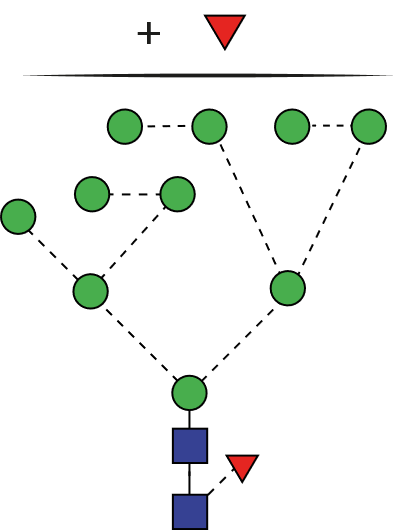 |
| **G10** | **779.90741** | **3** | **2342.74575** | **H6N3F2S1Phos2** | **[C_83_H_138_N_5_O_67_P_2_]^3-^** | 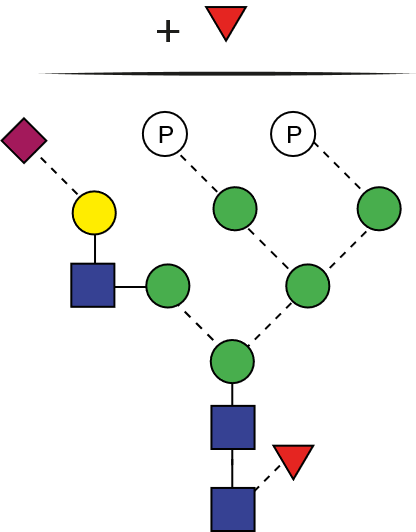 |
| **G11** | **785.00002** | **4** | **3144.03144** | **H7N4F3S2[G3S]2** | **[C_114_H_185_N_7_O_89_S_2_]^4-^** | **Not defined** |
| **G12** | **788.61107** | **3** | **2368.85673** | **H5N4F1S2** | **[C_90_H_145_N_6_O_66_]^3-^** | 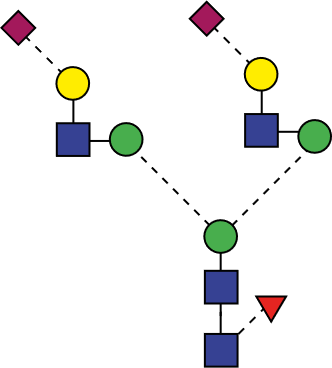 |
| **G13** | **815.26194** | **3** | **2448.80934** | **H5N4F1S2[G3S]1** | **[C_90_H_145_N_6_O_69_S_1_]^3-^** | 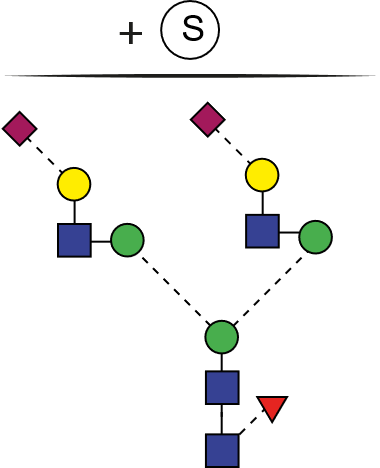 |
| **G14** | **824.27098** | **5** | **4126.3941** | **H8N7F1S4[G3S]** | **[C_154_H_246_N_11_O_115_S]^5-^** | 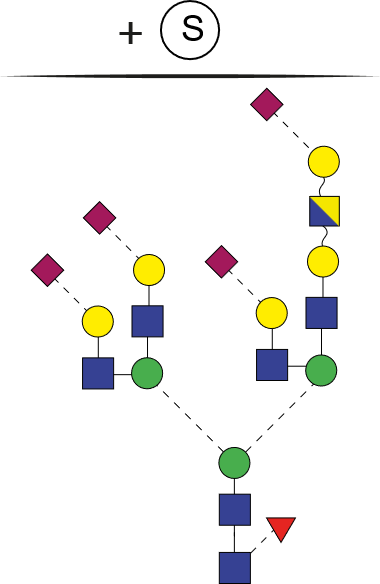 |
| **G15** | **828.03575** | **4** | **3316.17436** | **H6N5F1S4** | **[C_126_H_201_N_9_O_92_]^4-^** | 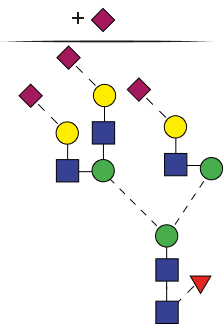 |
| **G16** | **848.02384** | **4** | **3396.12672** | **H6N5F1S4[G3S]1** | **[C_126_H_201_N_9_O_95_S]^4-^** | 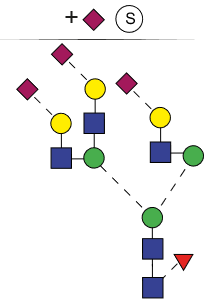 |
| **G17** | **853.52232** | **4** | **3418.12064** | **H9N5S2[G3S]1P2** | **[C_126_H_199_N_7_O_98_S]^4-^** | **Not defined** |
| **G18** | **856.30332** | **3** | **2571.93348** | **H5N5F1S2** | **[C_98_H_158_N_7_O_71_]^3-^** | 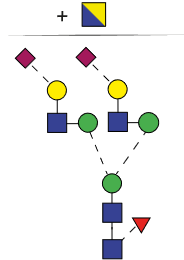 |
| **G19** | **866.53442** | **4** | **3470.16904** | **H7N6F1S3[G3S]1** | **[C_129_H_207_N_9_O_97_S]^4-^** | 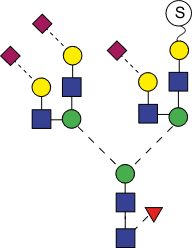 |
| **G20** | **876.791** | **4** | **3511.19536** | **H6N7F1S3[G3S]1** | **[C_131_H_214_N_10_O_97_S]^4-^** | 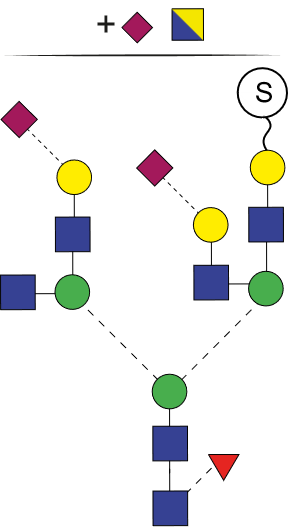 |
| **G21** | **882.80574** | **4** | **3535.25432** | **H7N6S4** | **[C_134_H_218_N_10_O_98_]^4-^** | 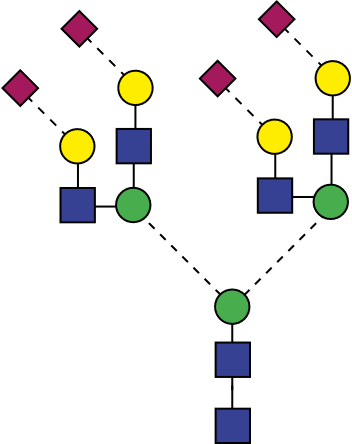 |
| **G22** | **885.64307** | **3** | **2659.95273** | **H5N4F1S3** | **[C_101_H_162_N_7_O_74_]^3-^** | 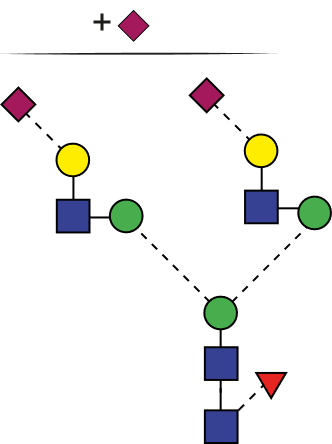 |
| **G23** | **890.96981** | **3** | **2675.93295** | **H6N4S3** | **[C_101_H_162_N_7_O_75_]^3-^** | 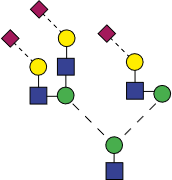 |
| **G24** | **910.32156** | **3** | **2733.9882** | **H6N5F1S2** | **[C_104_H_168_N_7_O_76_]^3-^** | 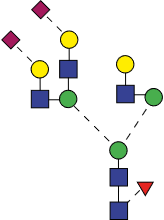 |
| **G25** | **919.31983** | **4** | **3681.31068** | **H7N6F1S4** | **[C_140_H_224_N_10_O_102_]^4-^** | 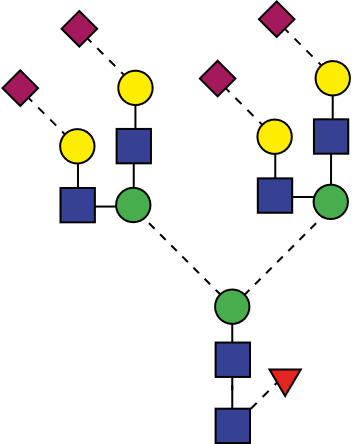 |
| **G26** | **936.97316** | **3** | **2813.943** | **H6N5F1S2[G3S]1** | **[C_104_H_168_N_7_O_79_S]^3-^** | 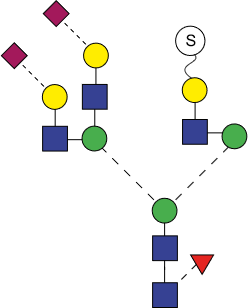 |
| **G27** | **958.66688** | **3** | **2879.02416** | **H6N5S3** | **[C_109_H_175_N_8_O_80_]^3-^** | 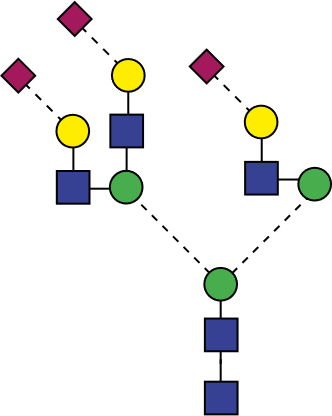 |
| **G28** | **974.08855** | **4** | **3900.38556** | **H8N7S4** | **[C_148_H_237_N_11_O_108_]^4-^** | 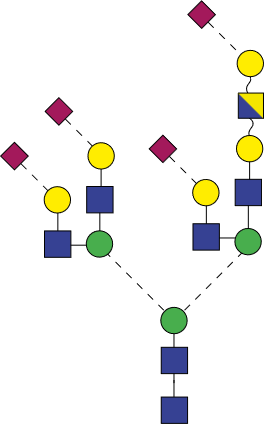 |
| **G29** | **978.01437** | **3** | **2937.06663** | **H6N6F1S2** | **[C_112_H_181_N_8_O_81_]^3-^** | 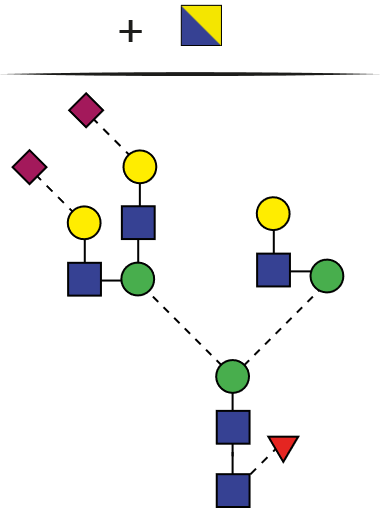 |
| **G30** | **1007.3527** | **3** | **3025.08162** | **H6N5F1S3** | **[C_115_H_185_N_8_O_84_]^3-^** | 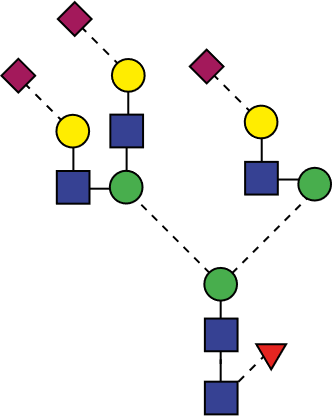 |
| **G31** | **1010.60168** | **4** | **4046.43808** | **H8N7F1S4** | **[C_154_H_247_N_11_O_112_]^4-^** | 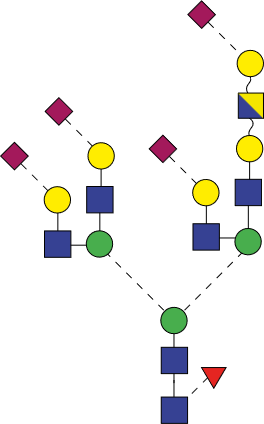 |
| **G32** | **1021.02653** | **3** | **3066.10311** | **H5N6F1S3** | **[C_117_H_188_N_9_O_84_]^3-^** | 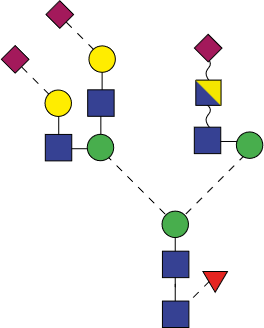 |
| **G33** | **1032.03187** | **3** | **3099.11913** | **H7N6F1S2** | **[C_118_H_191_N_8_O_86_]^3-^** | 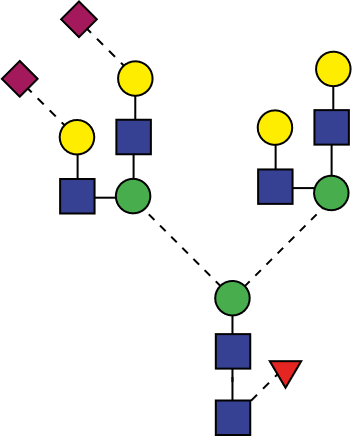 |
| **G34** | **1037.87266** | **2** | **2077.761** | **H5N4F1S1** | **[C_79_H_129_N_5_O_58_]^2-^** | 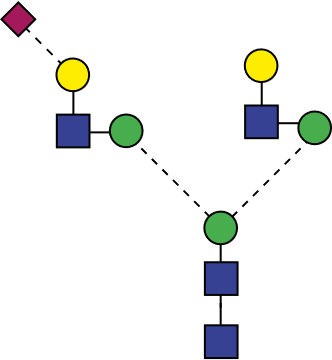 |
| **G35** | **1041.32848** | **3** | **3105.02703** | **H6N5F1S3[G3S]** | **[C_115_H_184_N_8_NaO_87_S]^3-^** | 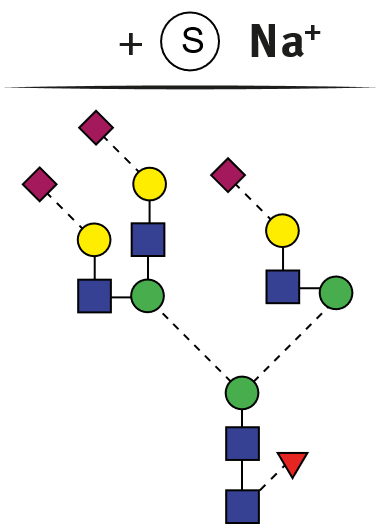 |
| **G36** | **1045.69737** | **3** | **3140.11563** | **H6N7F1S2** | **[C_120_H_194_N_9_O_86_]^3-^** | 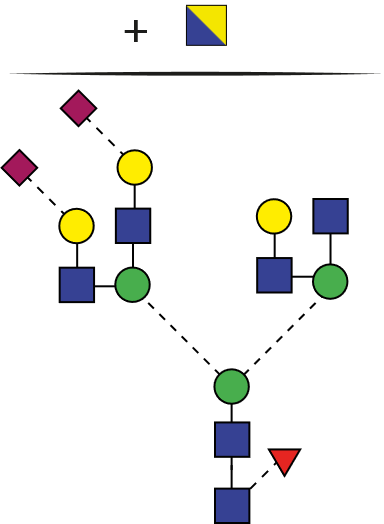 |
| **G37** | **1047.11844** | **4** | **4192.50512** | **H8N7F2S4** Stroop C et al Glycobiology,  2000 vol.10:901-917 | **[C_160_H_257_N_11_O_116_]^4-^** | 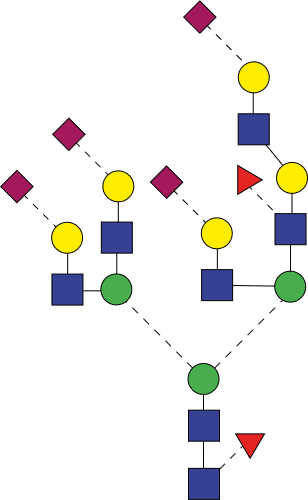 |
| **G38** | **1056.03946** | **3** | **3171.1419** | **H6N5F2S3** | **[C_121_H_195_N_8_O_88_]^3-^** | 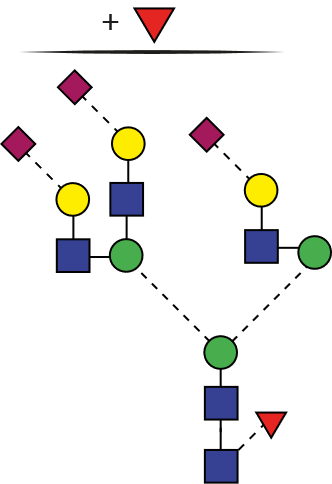 |
| **G39** | **1075.0464** | **3** | **3228.16272** | **H6N6F1S3** | **[C_123_H_198_N_9_O_89_]^3-^** | 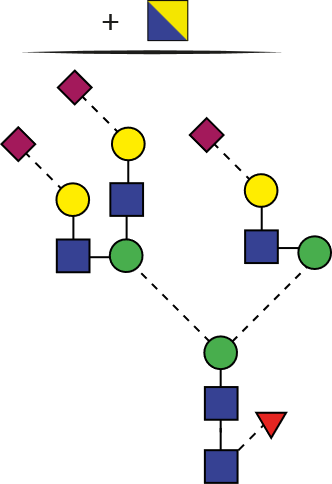 |
| **G40** | **1080.37949** | **3** | **3244.16199** | **H7N6S3** | **[C_123_H_198_N_9_O_90_]^3-^** | 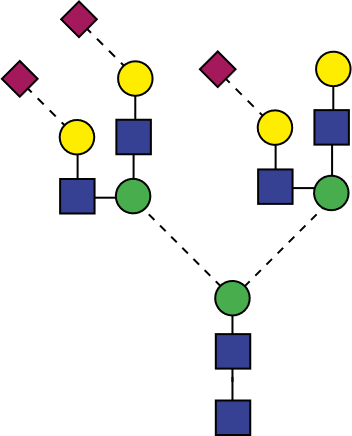 |
| **G41** | **1098.36191** | **2** | **2198.7395** | **H4N5F1S1[G3S]1** | **[C_81_H_132_N_6_O_61_S]^2-^** | 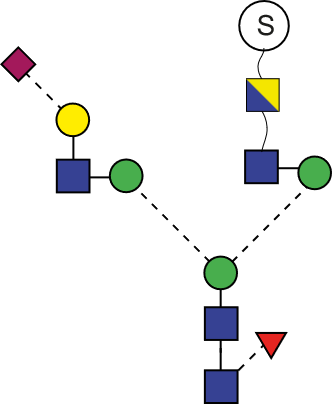 |
| **G42** | **1101.88613** | **4** | **4411.57588** | **H9N8F1S4** | **[C_168_H_270_N_12_O_122_]^4-^** | 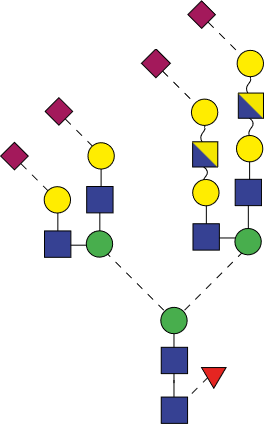 |
| **G43** | **1104.72157** | **3** | **3317.18823** | **H6N5F3S3** | **[C_127_H_205_N_8_O_92_]^3-^** | 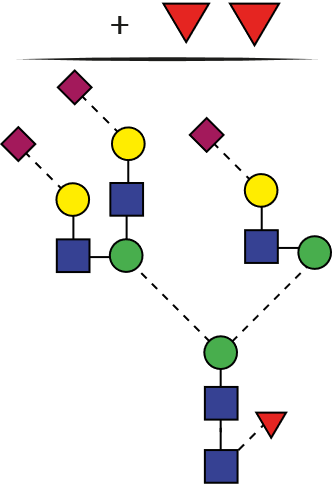 |
| **G5** | **1110.38993** | **2** | **2222.79554** | **H5N4S2** | **[C_84_H_136_N_6_O_62_]^2-^** | 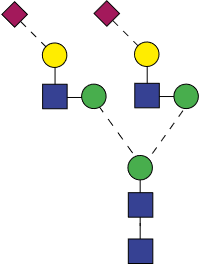 |
| **G5** | **1121.38364** | **2** | **2222.79554** | **H5N4S2** | **[C_84_H_135_N_6_NaO_62_]^2-^** | 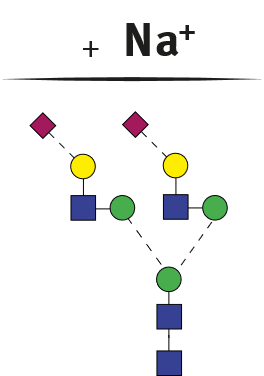 |
| **G44** | **1129.06391** | **3** | **3390.21525** | **H7N6F1S3** | **[C_129_H_208_N_9_O_94_]^3-^** | 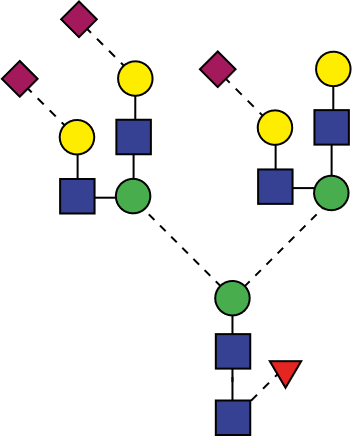 |
| **G9** | **1167.40457** | **2** | **2336.82482** | **H10N2F2** | **[C_88_H_146_N_2_O_69_]^2-^** | 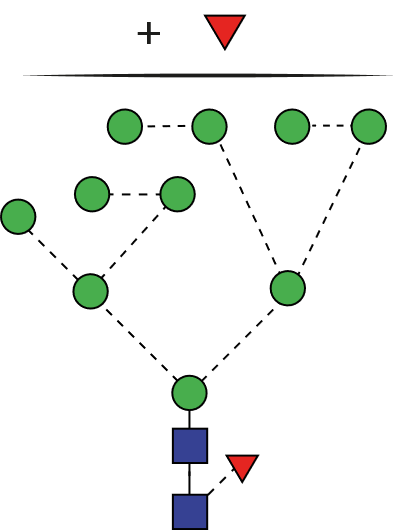 |
| **G21** | **1177.41163** | **3** | **3535.25841** | **H7N6S4** | **[C_134_H_215_N_10_O_98_]^3-^** | 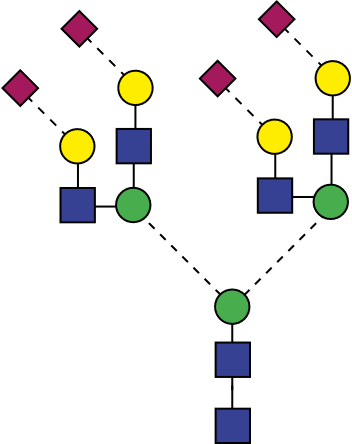 |
| **G12** | **1183.41997** | **2** | **2368.85562** | **H5N4F1S2** | **[C_90_H_146_N_6_O_66_]^2-^** | 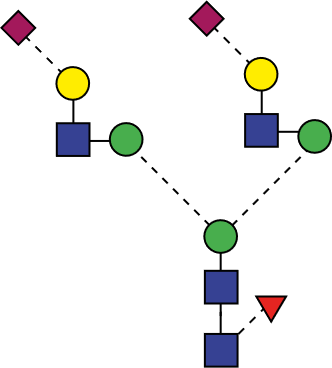 |
| **G45** | **1193.17038** | **4** | **4776.71288** | **H10N9F1S4** | **[C_182_H_293_N_13_O_132_]^4-^** | 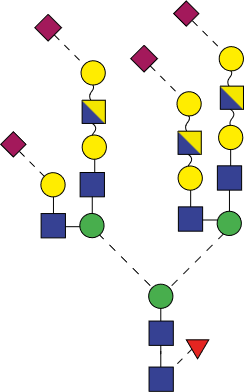 |
| **G46** | **1196.75691** | **3** | **3593.29425** | **H7N7F1S3** | **[C_137_H_221_N_10_O_99_]^3-^** | 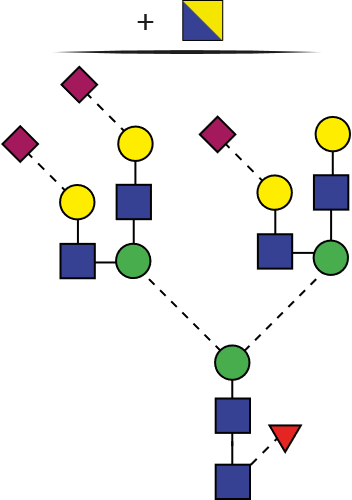 |
| **G47** | **1203.93124** | **2** | **2409.87816** | **H4N5F1S2** | **[C_92_H_149_N_7_O_66_]^2-^** | 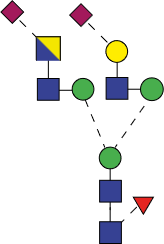 |
| **G25** | **1226.09402** | **3** | **3681.30558** | **H7N6F1S4** | **[C_140_H_225_N_10_O_102_]^3-^** | 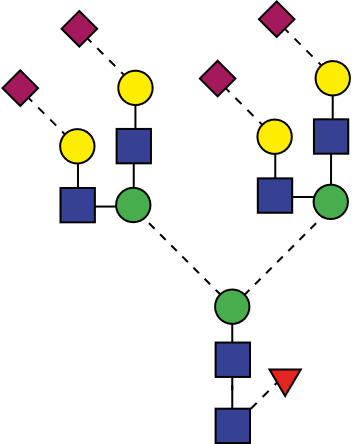 |
| **G49** | **1250.77478** | **3** | **3755.34786** | **H8N7F1S3** | **[C_143_H_231_N_10_O_104_]^3-^** | 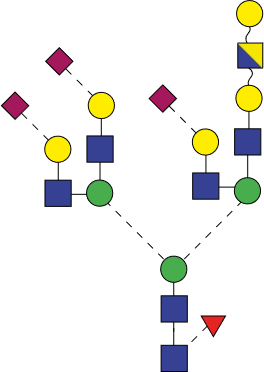 |
| **G50** | **1284.95876** | **2** | **2571.9332** | **H5N5F1S2** | **[C_98_H_159_N_7_O_71_]^2-^** | 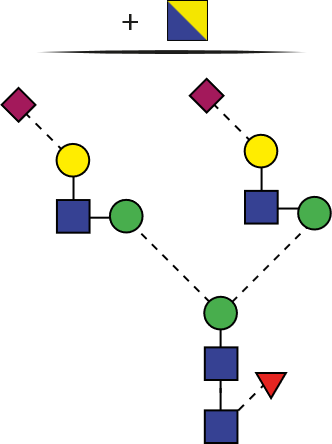 |
| **G51** | **1292.95909** | **2** | **2587.93386** | **H6N5S2** | **[C_98_H_159_N_7_O_72_]^2-^** | 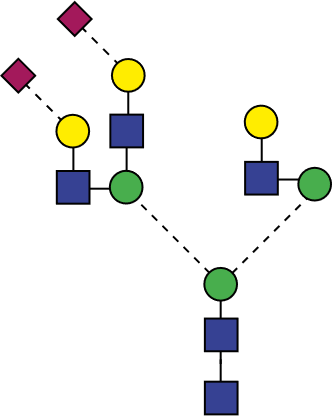 |
| **G52** | **1299.45919** | **3** | **3901.40109** | **H8N7F2S3** | **[C_149_H_241_N_10_O_108_]^3-^** | 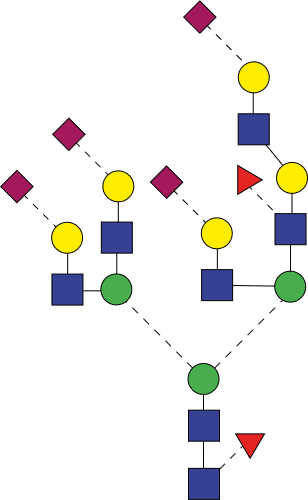 |
| **G24** | **1365.97928** | **2** | **2733.97424** | **H6N5F1S2** | **[C_104_H_169_N_7_O_76_]^2-^** | 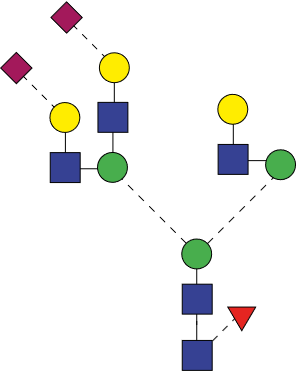 |
| **G53** | **1372.48713** | **3** | **4120.48491** | **H9N8F1S3** | **[C_157_H_254_N_11_O_114_]^3-^** | 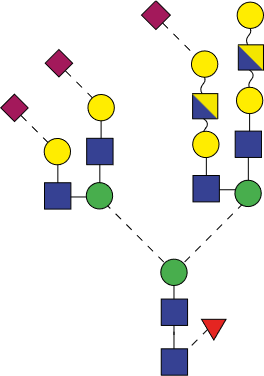 |
| **G54** | **1408.41855** | **2** | **2818.85278** | **unknown** | **[C_102_H_164_N_6_O_80_S_2_]^2-^** | **Not defined** |
